# Hepatitis B virus X protein upregulates HSP90alpha expression via activation of c-Myc in human hepatocarcinoma cell line, HepG2

**DOI:** 10.1186/1743-422X-7-45

**Published:** 2010-02-20

**Authors:** Weihua Li, Xiaohui Miao, Zhongtian Qi, Wenting Zeng, Jianxin Liang, Zengwei Liang

**Affiliations:** 1 Department of Infectious disease, the First affiliated Hospital of Guangzhou Medical College, Guangzhou 510102, Guangdong province, PR China; 2 Department of Infectious disease, the Second affiliated Hospital of Second Military Medical University, Shanghai 200433, PR China; 3 Department of Microbiology, Second Military Medical University, Shanghai 200441, PR China

## Abstract

**Background:**

The Hepatitis B Virus X protein (HBx) plays a major role in hepatocellular carcinoma (HCC) development, however, its contribution to tumor invasion and metastasis has not been established so far. Heat shock protein 90 alpha (HSP90alpha) isoform is an ATP-dependent molecular chaperone that maintains the active conformation of client oncoproteins in cancer cells, which is abundantly expressed in HCC, especially in hepatitis B virus (HBV)-related tumors, might be involved in tumor progression.

**Methods:**

The levels of HSP90alpha, extracellular signal-regulated kinase 1/2 (ERK1/2), phosphorylated ERK1/2 (p-ERK1/2) and c-Myc in HBx-transfected HepG2 cells were determined by western blots analysis. The endogenous ERKs activity was demonstrated by ELISA assay. The regulation of c-Myc-mediated HSP90 alpha promoter transactivation by HBx was evaluated through electrophoretic mobility shift analysis (EMSA). The c-Myc-mediated HSP90alpha transcription was analysed by promoter assay. The HBx-expressing cells were transfected with specific small interference RNA (siRNA) against c-Myc. The *in vitro *invasion potentials of cells were evaluated by Transwell cell invasion assay.

**Results:**

HBx induces HSP90alpha expression at the transcription level. The induction effect of HBx was inhibited after treatment with c-Myc inhibitor, 10058-F4. In addition, the luciferase activity of the HSP90alpha promoter analysis revealed that the HBx is directly involved in the c-Myc-mediated transcriptional activation of HSP90alpha. Furthermore, HBx induces c-Myc expression by activation of Ras/Raf/ERK1/2 cascades, which in turn results in activation of the c-Myc-mediated HSP90alpha promoter and subsequently up-regulation of the HSP90alpha expression. Overexpression of HSP90alpha in HBx-transfected cells enhances tumor cells invasion. siRNA-mediated c-Myc knockdown in HBx-transfected cells significantly suppressed HSP90alpha expression and cells invasion *in vitro*.

**Conclusion:**

These results demonstrate the ability of HBx to promote tumor cells invasion by a mechanism involving the up-regulation of HSP90alpha and provide new insights into the mechanism of action of HBx and its involvement in tumor metastasis and recurrence of HCC.

## Background

Hepatitis B virus (HBV) is strongly associated with the development of hepatocellular carcinoma (HCC) [1]. One of the open-reading frames encoded by the HBV genome is an oncogenic X protein (HBx), which is the most frequently integrated viral sequence found in HCCs. HBx is likely to be implicated in the several different steps of carcinoma development. Most efforts in the study of the role of HBx in HCC development have focused on its involvement in the genesis of liver carcinomas. In this regard, HBx is able to induce HCC either alone or in synergy with c-Myc or chemical carcinogens in transgenic mice [2,3]. Although it does not bind directly to DNA, HBx affects transcriptional activation via its interaction with nuclear transcription factors and the cytoplasmic modulation of signal transduction pathways. HBx activates several signal transduction pathways that lead to the transcriptional upregulation of a number of cellular genes, including those of growth factors and oncogenes [4]. In addition, HBx promotes cell cycle progression, inactivates negative growth regulators like p53 [5] and facilitates the accumulation of DNA mutations by interfering with the DNA repair machinery [6]. HBx is also able to interfere with apoptotic signals, leading to tumor cell survival, although this issue remains controversial [7].

Recently, several reports have demonstrated that HBx is also implicated in the late stages of tumor progression, metastasis and angiogenesis. HBx induces extensive morphological changes and cytoskeleton rearrangements in liver cells [8]. It induces adherens junction disruption [9] and modulates integrin-mediated adhesion to extracellular matrix (ECM) [10]. In addition, HBx promotes tumor cell invasion by inducing membrane-type matrix metalloproteinase 1 (MT1-MMP) [11], MMP-9 [12] and urokinase-type plasminogen activator [13] or reducing E-cadherin [14]. Also, HBx activates hypoxia-inducible factor-1a, which promotes angiogenesis through activation of several angiogenic factors like vascular endothelial growth factor [15]. However, the role of HBx in tumor invasion and metastasis and the underlying mechanisms are far from being fully understood.

Heat shock protein 90 (HSP90) is an important molecular chaperone and have key roles in signal transduction, protein folding, protein degradation, and morphological evolution [16]. The HSP90 is abundantly expressed by a variety of tumor types and has been recently targeted for cancer therapy [17]. Recently, several reports have shown that HSP90alpha isoform is associated with the invasive and metastatic abilities of the human breast cancer cells. Cell-surface HSP90alpha is involved in heregulin-induced the receptor tyrosine kinase HER-2 activation and signaling, leading to cytoskeletal rearrangement, essential for cell invasion [18,19]. The extracellular hyperacetylated HSP90alpha promotes extracellular maturation of MMP-2, involved in tumor invasion and metastasis [20,21]. As a result of this the overexpression of HSP90alpha is common in various human tumours, preferentially in malignant cancers, correlates with poor prognosis and resistance to therapy [22]. Owing to its key role in the activity of various oncogenic proteins and pathways, HSP90alpha may play a unique role in tumor metastasis. Immunohistochemical studies of HSP90alpha expression in HBV-related HCC demonstrated significant upregulation of HSP90alpha in the tumor tissue compared with adjacent nontumor tissues [23]. Recently, we have shown that HSP90alpha is upregulated in the HBxpositive HepG2 cells compared with HBx-negative HepG2 cells by proteomic analysis [24]. However, the underlying mechanism for the HSP90alpha activition in HBV induced HCC is still unknown.

In this study, we demonstrate that HBx induces c-Myc expression by activation of Ras/Raf/ERK1/2 pathway, which in turn results in activation of the c-Myc-mediated HSP90alpha promoter and subsequently up-regulation of the HSP90alpha expression. Moreover, Overexpression of HSP90alpha in HBx-transfected cells enhances tumor cells invasion. Repression of endogenous c-Myc expression by siRNA significantly reduces HSP90alpha expression and the invasive capacity of HBx-transfected cells. These results demonstrate that HBx has an effect on the up-regulation of the HSP90 alpha, which is associated with tumor cells invasion and support a role for HBx in the late steps of tumor development and metastasis.

## Results

### HBx upregulates HSP90alpha expression

**Figure 1:**
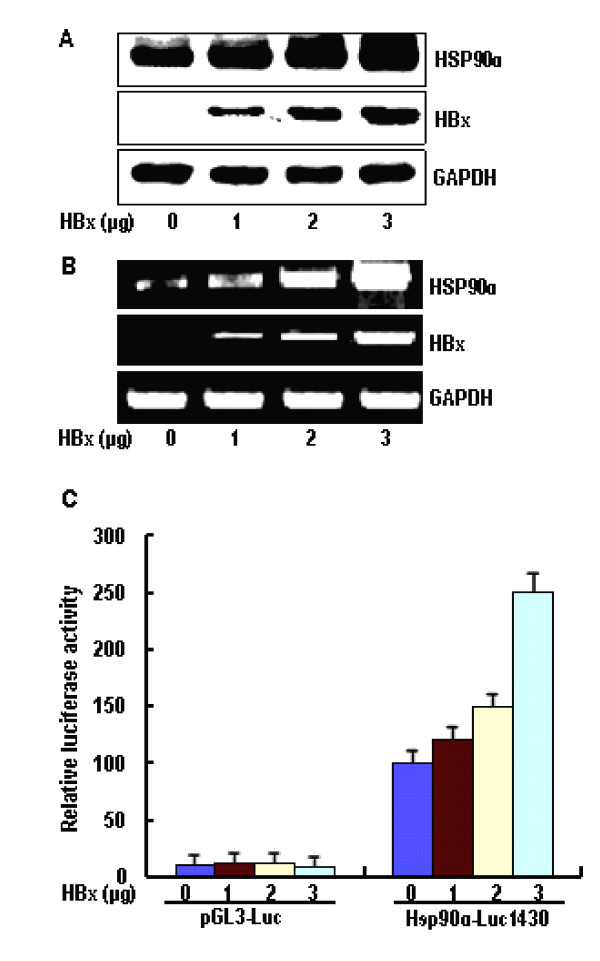
**Upregulation of Hsp90α expression by HBx**. (A) HepG2 cells were transiently transfected with the indicated amount (1 μg, 2 μg, 3 μg) of pcDNA3-X and Western blotting of HSP90α was performed. (B) Total RNA purified from the HBxtransfected HepG2 cells was subjected to RT-PCR for measuring HSP90α transcripts. (C) Increasing amount (1 μg, 2 μg, 3 μg) of pcDNA3-X was cotransfected with 2 μg of HSP90α promoter-luciferase reporter constructs (Hsp90α-Luc1430), and 2 μg of β-galactosidase reporter plasmid into HepG2 cells and luciferase assay was performed. Luciferase activity was normalized with the β-alactosidase activity in cell lysate. Error bars indicate standard deviations (SD) obtained from three different experiments prepared in triplicate.

The up-regulation of HSP90alpha is implicated in the malignant progression of HCCs, especially in HBV-related tumors [16,23]. However, the viral factor responsible for this phenomenon is unknown. Therefore, we first investigated whether HSP90alpha expression is activated by HBx in cultured human liver cells. For this purpose, we transiently transfected HBxexpressing plasmid, pcDNA3-X, into a human hepatoma cell line HepG2. As a result, the HSP90alpha protein level was increased by HBx in a dose-dependent manner, as determined by Western blot analyses (Figure 1A). The level of HSP90alpha mRNA in HepG2-pcDNA3-X cells, as determined by semiquantitative RT-PCR, was similarly increased by HBx (Figure 1B), which suggests that HBx increases HSP90alpha expression at the transcription level. Furthermore, to investigate the effect of HBx on the HSP90alpha promoter activity, we performed luciferase assay, using the HSP90alpha-luciferase reporter (HSP90α-Luc1430) that contains luciferase gene under the control of full-length HSP90alpha promoter. As a result, we found that HBx specifically activates the HSP90alpha promoter activity in a dose-dependent manner, up to approx ~2.5-fold (Figure 1C). Therefore, we confirm that HBx upregulates HSP90alpha expression at the transcription initiation step by modulating its promoter activity.

### HBx upregulates HSP90alpha expression by activating c-Myc

**Figure 2:**
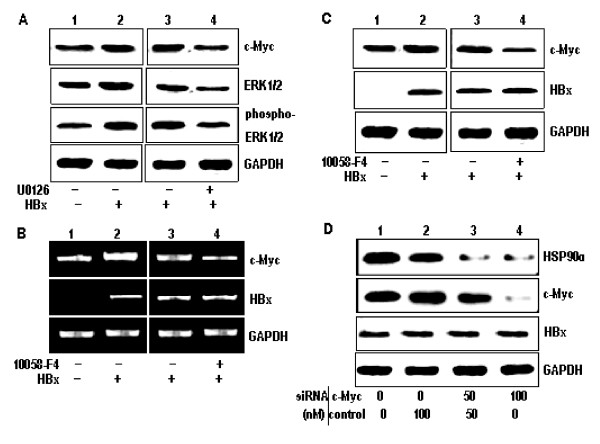
**HBx upregulates Hsp90α expression by activating c-Myc**. (A) Lysates from stable cell lines, HepG2-pcDNA3 (lane 1) and HepG2-pcDNA3-X (lanes 2-4) after either mock treatment (lanes 1-3) or treatment with 200 nM U0126 (lane 4) for 4 h were prepared, and the protein levels of c-Myc, ERK1/2 and phosphor-ERK-1/2 were detected by Western blot analysis. GAPDH was included as an internal control. (B) Total RNA purified from HepG2-pcDNA3 (lane 1) and HepG2-pcDNA3-X (lanes 2-4) after either mock treatment (lanes 1-3) or treatment with 5 mM 10058-F4 (lane 4) for 24 h was subjected to RT-PCR to measure the RNA level of c-Myc, HBx and GAPDH. (C) Western blot analysis was performed to measure the levels of c-Myc, HBx and GAPDH in the cells prepared as described above. (D) HepG2-pcDNA3-X cells were transfected with 100 nM of either control siRNA, c-Myc siRNA or in a combination and Western blotting analysis was performed.

It is well known that HBx mediates the activation of signal transduction pathways such as the Ras/Raf/ERK1/2 cascades, leading to the induction of c-myc [25]. To investigate HBx activates c-Myc expression via the Ras/Raf/ERK1/2 pathway, we first detected the protein levels of c-Myc, ERK1/2 and phosphorylation of ERK-1/2 in HepG2-pcDNA3 and HepG2-pcDNA3-X cells by western blot analysis. As shown in Figure 2A, the expression of c-Myc, ERK1/2 and phosphorylation of ERK-1/2 were increased in the HepG2-pcDNA3-X cells was compared with HepG2-pcDNA3 cells. Futhermore, the c-Myc, ERK1/2 and phosphorylation of ERK-1/2 were decreased in HepG2-pcDNA3-X cells treated with U0126, a known ERKs inhibitor (Figure 2A). These results show that HBx activates the c-Myc protein through the Ras/Raf/ERK1/2 pathway.

It is reported that the upstream portion of the HSP90alpha gene contains a c-Myc binding element, which this element is required for the inducible transactivation of HSP90alpha by c-Myc, and that HSP90alpha mRNA and protein levels are also elevated in response to c-Myc induction [26]. Therefore, we next tried to investigate whether the up-regulation of HSP90alpha expression in the HBx-expressing cells is due to enhanced expression of c-Myc or not. Both mRNA and protein levels of c-Myc were significantly increased in the HBx-expressing cells (Figure 2B and 2C, respectively), which suggests that HBx induces the expression of c-Myc by stimulating its transcription. Treatment of the HBx-expressing cells with the c-Mycspecific inhibitor 10058-F4 resulted in a significantly decreased of both mRNA and protein levels of HSP90alpha (Figure 2B and 2C), suggesting that 10058-F4 blocked the induction of HSP90alpha activation by HBx.

To provide a more direct evidence for the role of c-Myc in the regulation of HSP90alpha gene by HBx, we attempted to knock down c-Myc expression by introducing a specific siRNA into HBx-expressing cells. As expected, c-Myc expression was specifically decreased by c-Myc siRNA in a dose-dependent manner (Figure 2D). Accordingly, HSP90alpha expression was decreased, proving that c-Myc plays a direct role in the induction of HSP90alpha expression by HBx.

### Transactivation of the HSP90alpha promoter by HBx through the c-Myc-binding site

**Figure 3:**
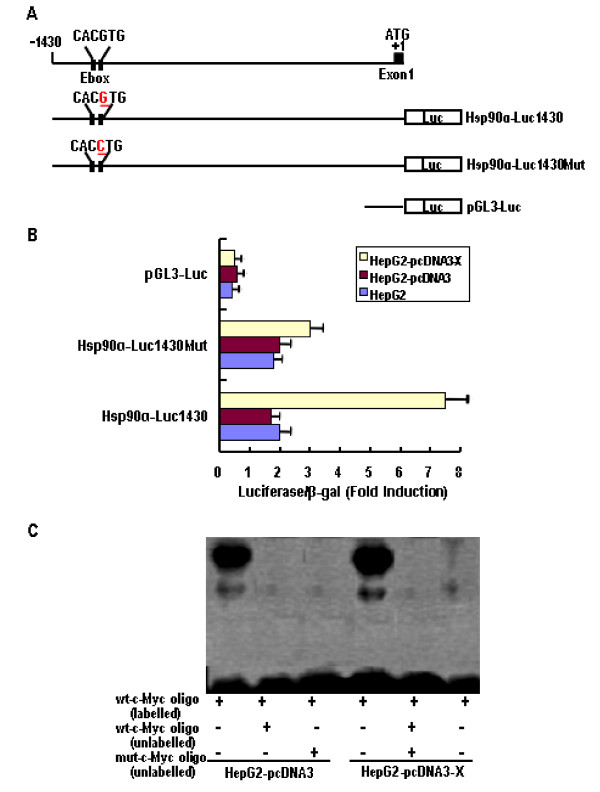
**Regulation of c-Myc-mediated Hsp90α promoter transactivation by HBx**. (A) Schematic illustration of the genomic region encompassing the 5' flanking region of the human the Hsp90α promoter and the reporter constructs used. (B) Cells were cotransfected with or without 1 μg of pcDNA3 or pcDNA3-X plasmid, 2 μg of Hsp90α promoter-luciferase reporter construct, and and 2 μg of β-galactosidase reporter plasmid by the LipofecAMINE method. Cells were cultured in 10% FBS medium for 24 h. Luciferase activity and β-galactosidase activity were assayed by using the luciferase and β-galactosidase enzyme assay system. Luciferase activity was normalized with the β-galactosidase activity in cell lysates. Error bars indicate standard deviations (SD) obtained from three different experiments prepared in triplicate. (C) The nuclear extract isolated from HepG2-pcDNA3 (lane 1-3) and HepG2-pcDNA3-X (lanes 4-6) cells incubated with [γ^-32^P]ATP-labeled oligonucleotides corresponding to the wt c-Myc binding site of the Hsp90α promoter. Competition was performed using unlabeled wt oligo or mutant oligo. Each sample was electrophoresed in a 4% nondenaturing polyacrylamide gel in 0.5 × Tris-borate EDTA buffer at 250 V for 20 min. The gel was dried and subjected to autoradiography.

To investigate whether the transcriptional activity of HSP90alpha is regulated by HBx through the c-Myc binding site, the promoter activity of the HSP90alpha gene was examined. As shown in Figure 3A, we constructed a reporter vector (HSP90 α-Luc1430)containing segments of the 5' flanking region and part of the exon 1 of the HSP90alpha gene, including an E-box site (CACGTG) in the proximal promoter, linked to the promoter coding domain of the luciferase reporter gene (pGL3-luc). In addition, to determine whether the E-box site (CACGTG) was responsible for c-Mycmediated activation, its sequence was changed from CACGTG to CACCTG using site directed mutagenesis, a reporter vector (HSP90alpha-Luc1430Mut) was constructed. These plasmids were cotransfected into HepG2 cells with the pcDNA3 or the pcDNA3-X vectors. As shown in Figure 3B, the luciferase activity of the HSP90alpha-Luc1430 cotransfected with pcDNA3-X was ~5.0-fold higher than those for the pGL3-Luc and the HSP90alpha-Luc1430 cotransfected with pcDNA3 or without the vector. However, the luciferase activity remained essentially unchanged, when the pGL3-Luc and the HSP90alpha-Luc1430Mut were cotransfected with pcDNA3-X, pcDNA3, or without the vector into HepG2 cells, respectively. The results showed that the luciferase activity of the HSP90alpha promoter containing the c-Myc binding element was significantly increased in HBx-transfected cells, compared with HepG2 and HepG2-pcDNA3 cells, thus indicating that the E-box in the promoter region of the HSP90alpha gene mediates transcriptional activation by c-Myc.

To additionally confirm that HBx is directly involved in the c-Myc-mediated transcriptional activation of HSP90alpha, we examined whether HBx is correlated to the binding of c-Myc to wild-type oligonucleotides that contain the sequence for the c-Myc binding site from the HSP90alpha promoter by EMSA or not. As shown in Figure 3C, we confirmed that the nuclear extract isolated from HepG2-pcDNA3 and HepG2-pcDNA3-X cells all induced an electromobility shift when a labeled wild-type oligonucleotide was introduced. In addition, the intensity levels of the shifted bands in the nuclear lysates from HepG2-pcDNA3 cells were lower than those for the nuclear lysates from HepG2-pcDNA3-X cells. Moreover, the formation of an electrophoretically retarded complex was inhibited when an unlabeled wild-type oligonucleotide or mutant-type oligonucleotides was introduced. These results suggest that HBx regulates c-Myc-mediated transcription of the HSP90alpha gene.

### HSP90alpha up-regulation by HBx increases cellular invasion ability

**Figure 4:**
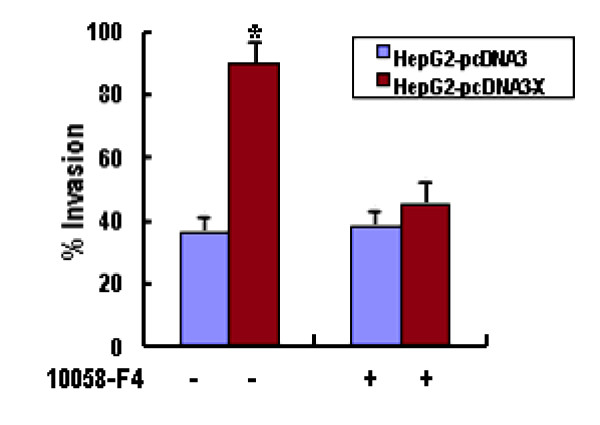
**HBx increases cell invasion ability**. Matrigel invasion assay of HepG2-pcDNA3 and HepG2-pcDNA3-X cells. Cells (1 × 10^6^) were resuspended in conditioned medium and added to the upper compartments of matrigel invasion chambers supplemented with or without 5 mM 10058-F4 for 24 h. The total number of cells that invaded to the underside of the filters was counted. The values obtained were calculated by averaging the total number of cells from three filters. As a control, a parallel experiment was performed using the device with uncoated membrane. The results represent means of triplicates; * statistically different from control at P < 0.05.

It has been described that increased HSP90alpha expression is strongly correlated with enhanced cell invasiveness in vitro and tumor progression in vivo [19-21]. Therefore, we finally examined whether the increased HSP90alpha by HBx affects cell invasiveness in vitro. Each cell line was either left untreated or treated with 10058-F4 for 24 h and subjected to the transwell invasion assay. As shown in Figure 4, the invasion capacity of HBxexpressing cells was increased when compared with HepG2 and HepG2-pcDNA3 cells. Moreover, the invasion ability of HBx-expressing cells was dramatically decreased in the presence of 10058-F4. Therefore, we prove that HBx-mediated HSP90alpha up-regulation increases cell invasion ability in vitro.

## Discussion

HSP90 is of interest because of its importance in maintaining the conformation, stability and function of key oncogenic proteins involved in signal transduction pathways leading to proliferation, cell cycle progression and apoptosis, as well as other features of the malignant phenotype such as invasion, angiogenesis and metastasis [27].

Several recent studies provided consistent evidence for a role of HSP90alpha isoform in tumor invasive and metastatic phenotypes [19-22]. Transfection of HSP90alpha cDNA into invasive carcinoma cells led to significant enhancement of their invasive capacity in vitro [21], and activation of HSP90alpha resulted in increasing of malignant potentials of tumor cell lines [22]. In addition, up-expression of HSP90alpha during tumor development was observed in a variety of different tumor types, including HCC and is closely associated with a poor prognosis and resistance to therapy [28]. Therefore, knowledge of the molecular mechanism that activates its expression or function is of primary importance in understanding the process of tumor invasion. Here, we further demonstrate that HBx induces c-Myc expression by activation of Ras/Raf/ERK1/2 cascades, which in turn results in activation of the c-Myc-mediated HSP90alpha promoter and subsequently upregulation of the HSP90alpha expression, leading to the increased invasive potential of HBx-expressing cells.

It is well known that HBx mediates the activation of signal transduction pathways such as the Ras/Raf/ERK1/2 cascades, leading to the induction of c-myc [29]. The c-myc proto-oncogene is involved in the control of cell cycle progression, proliferation, metabolism, and apoptosis [30]. c-Myc protein is a transcription factor that functions via heterodimerization with MAX, a related protein that, like c-Myc, contains basic, helix-loop-helix and leucine zipper domains but lacks the transactivation domain present in the amino terminus of c-Myc [31]. MYC/MAX complexes stimulate transcription of target genes containing the MYC/MAX binding site (E-box) or specific non-canonical elements in their regulatory regions [32]. The c-myc proto-oncogene encodes a ubiquitous transcription factor involved in the control of cell growth and differentiation and is implicated in inducing hepatocarcinoma tumourigenesis [33]. Understanding the function of c-myc and its role in cancer depends upon the identification of c-myc target genes. The findings of Teng et al [26] that c-myc directly activates HSP90alpha transcription suggest that by induction of HSP90alpha c-myc may control the activity of multiple signal pathways involved in cellular transformation. Recently, several studies showed that the increased transcription of HSP90alpha in tumour cells is due to higher expression of the protooncogenes HER2, c-Myc, k-ras and other genes is crucial to tumourigenesis [34,35]. Although HBx has been reported to be associated with HCC, there is no confirmative report of transcription factor regulating expression of HSP90alpha by HBx, which is related to invasion and metastasis of HCC. The promoter region of HSP90alpha gene has a c-Myc binding site and plays an important role in HSP90alpha gene activation. Thus, it is possible to speculate that HBx up-regulates HSP90alpha expression by elevating the activity of transcriptional factor c-Myc.

The findings presented here clearly show that HBx up-regulates HSP90alpha expression by inducing the expression of c-Myc in HBx-transfected cells that express HBx transcripts (Figure 2A and 2B). Moreover, the increased expression of HSP90alpha in the presence of HBx could be completely inhibited by treatment with c-Myc inhibitor 10058-F4 or introducing a specific-siRNA (Figure 2C).

Teng et al [26] reported that there is an E-box site (CACGTG) in the 5' promoter of HSP90alpha gene that binds c-Myc, which is located the DNA sequence between bases -1104 and -998, and that the HSP90alpha promoter-derived oligonucleotide can specifically bind to c-Myc, as assayed by EMSA. In addition, the mutated HSP90alpha promoter, in which the E-box is destructed by point mutations by changing the DNA sequence from CACGTG to CACCTG in c-Myc binding site of the HSP90alpha promoter, showed affect on transactivation of c-Myc and loss response to HBx with the wild-type promoter, as measured by a luciferase reporter assay. Furthermore, HSP90alpha mRNA and protein levels are elevated in response to c-Myc induction in HBx-transfected cells (Figure 1A and 1B). HBx is known to activate c-Myc transcriptional activity through ERK1/2. Therefore, it is possible to speculate that HBx may activate the HSP90alpha gene via up-regulation of c-Myc activity since HSP90alpha promoter contains the binding motifs of the c-Myc complex.

In this study, we observed that overexpression of HSP90alpha enhanced invasive activity of HBx-expressing cells (Figure 4), demonstrating the oncogenic property of HSP90alpha when its expression is increased. This upregulation of the metastatic abilities of tumor cells was corroborated by the Matrigel invasion assays, in which HBx-expressing cells also displayed increased invasive potential. In addition, treatment with c-Myc inhibitor 10058-F4 or siRNA experiments to repress the endogenous HSP90alpha levels in HBx-expressing cells decreased their invasion activity (Figure 4). These results are consistent with the role of increased HSP90alpha levels by HBx contributing to malignant phenotype.

## Conclusion

In summary, we conclude that HBx up-regulates HSP90alpha expression by inducing the c-Myc-mediated HSP90alpha promoter activation. The incressed HSP90alpha expression results in inducing cell invasion of the HBx-expressing cells. In addition, Repression of endogenous c-Myc expression by siRNA significantly reduces HSP90alpha expression and the invasive capacity of HBx-expressing cells. Therefore, the present study suggests that HBx plays a role during the late steps of tumor development and metastasis by increasing invasion ability from the surrounding cells and migration outside of the primary tumor site. This study provides a new clue for understanding the role of HBx during HCC progression, unveiling potential new target in the therapy against HBV-related HCC.

## Materials and methods

### Cell culture

Human hepatocarcinoma cell lines, HepG2, were obtained from Foundation Institute, Chinese Academy of Medical Sciences. Stable cell lines, HepG2-pcDNA3 and HepG2-pcDNA3-X, were established by transfection with either an empty vector pcDNA3 or pcDNA3-X encoding the corresponding full-length HBx sequence (nt1374-1838) as described previously (24), followed by selection with 500 mg/ml G418 (Gibco-BRL, NY, USA). All cells were cultured in Dulbecco's modified Eagle's medium (DMEM, Gibco-BRL) supplemented with 10% fetal bovine serum (FBS), 100 U/ml penicillin and 100 μg/ml streptomycin, 5 mmol/L L-glutamine and 200 μg/ml G418 at 37°C in a humidified chamber with 5% CO2.

### Semiquantitative RT-PCR

Total cellular RNA was extracted from HepG2 cells using Trizol (Invitrogen). DNase I (Promega)-digested RNA (2 μg) was reverse transcribed with the corresponding antisense primer. One-quarter of the reversetranscribed RNA was amplified with Taq DNA polymerase (Promega) (94°C for 2 min; 35 cycles of 94°C for 30 s, 58°C for 20 s, 72°C for 30 s, 72°C for 5 min) using the sense primers, 5'-AAACACCTGGAGATAAACCC-3', 5'-TTCGGGTAGTCGAAAACCAG-3', 5'-ACCGAATTCCCATGGCTGCT-3' and 5'-ACCACAGTCCATGCCATCAC-3'; and antisense primers, 5'-GTATCAT CAGCAGTAGGGTCA-3', 5'-CAGCAGCTCGAATTTCTTCC-3', 5'-AACTCTAGATGATTAGGCAGAGGT-3' and 5'-TACAGCAACAGGGTGGTGGA-3' for HSP90alpha, c-Myc, HBx and glyceraldehyde 3-phosphate dehydrogenase (GAPDH), respectively. PCR products were separated by 1.8% agarose gel electrophoresis, then scanned and analyzed by VDS imagemaster system (Pharmacia).

### Western blot analysis

Cells were homogenized in buffer containing 50 mM Tris-HCl (pH 8.0), 150 mM NaCl, 0.02% NaN_3_, 100 μg/mL PMSF, 1 μg/mL aprotinin, and 1% Triton X-100. Protein concentration of cell extracts was measured using the bovine serum albumin protein assay kit (Bio-Rad). In all, 20 μg of cell lysates were separated by SDS-polyacrylamide gel electrophoresis and transferred onto a nitrocellulose membrane (Hybond PVDF, Amersham). The membranes were blotted for 60 min at room temperature with non-fat dry milk (5%) in TBS containing 0.05% Tween-20 and were then incubated with specific primary antibodies: rabbit polyclonal anti-human HSP90alpha antibody (Cell Signaling Technology, USA, 1:1000), mouse monoclonal anti-human c-Myc antibody (Santa Cruz, USA, 1:1000.), mouse monoclonal antihuman HBx antibody (Roche, USA, 1:500), mouse monoclonal anti-GAPDH antibody (Sigma, USA, 1:1000) overnight at 4°C. Detection was performed using a secondary horseradish peroxidase-linked antimouse and rabbit antibody, and an enhanced chemiluminescence system (Amersham).

### ERK activity assay

ERK activation was determined with a fast-activated, cell-based, enzyme-linked immunosorbent assay kit (Active Motif) according to the manufacturer's instructions. Briefly, cultured cells placed in 96-well plates were further cultured for 3 days and then treated with or without U0126 (the ERKs inhibitor) (Calbiochem). The inhibitors were added 1 hour before stimulation with culture medium and at stimulation. At the indicated times, the cells were fixed with 4% formaldehyde for 20 minutes, extensively washed, and incubated with a specific antiphosphorylated ERK1/2 antibody, followed by incubation with a secondary horseradish peroxidase-conjugated antibody. Phosphorylated ERK1/2 levels were quantified with a colorimetric readout and are expressed as the absorbance at 450 nm measured in each well.

### RNA interference

Both c-Myc siRNA vector (pGB-c-Myc) and negative control siRNA vector (pGB-control) were purchased from BioVision Biotechnology. Cells (2 × 10^6^) were transfected with 100 nM of siRNA using Lipofectamine 2000 (Invitrogen) in Opti-MEM I reduced serum medium (Invitrogen) for 6 h. The medium was removed and replaced with fresh DMEM supplemented with 10% FBS serum. Cells were harvested 72 h after transfection for Western blotting analysis.

### Electrophoretic mobility shift assay (EMSA)

The nuclear extracts were prepared from treated and control HepG2-pcDNA3-X cells using a nuclear extraction kit (Active Motif). EMSA were performed using gel shift assay system kit (Promega) according to the manufacturer's instructions. Briefly, double-stranded oligonucleotides containing the consensus sequences 5'-GGGGCCCACGTGGCTGCTAGTTT-3' (wild-type HSP90alpha c-Myc binding site) were end-labeled with [γ^-32^P] ATP (3000 Ci/mmol; Amersham Pharmacia Biotech, UK) using T4 polynucleotide kinase and used as probes for EMSA. Competition was also performed using either the unlabeled wild-type oligonucleotide or a mutant oligonucleotide 5'-GGGGCCCACCTGGCTGCTAGTTT-3' (mutant oligo). Nuclear extract proteins (2 μg) were preincubated with the gel shift binding buffer [4% glycerol, 1 mM MgCl2, 0.5 mM EDTA, 0.5 mM dithiothreitol, 50 mM NaCl, 10 mM Tris-HCl (pH 7.5), and 0.05 mg/ml poly (deoxyinosine-deoxycytosine)] for 10 min and then incubated with the labeled probe for 20 min at room temperature. Each sample was electrophoresed in a 4% nondenaturing polyacrylamide gel in 0.5 × TBE buffer at 250V for 20 min. The gel was dried and subjected to autoradiography.

### Promoter assay

The genomic region flank of the HSP90alpha gene promoter (GenBank Accession No. U25822) was obtained from genomic DNA of HepG2 cells by PCR amplification using a primer set: 5'-GACGCTCGATGCTCGAGCCTGGGGGACCAAG-3' (forward/*Xho*I) and 5'-CGTTAAGCGCCTCCGCCCTGCACCCCCA-3' (reverse/*Hind*III), and the sequence was verified by sequencing. To generate the reporter construct driven by HSP90alpha promoter, a ~1.4-kb fragment containing 1430 bp 5' upstream of transcription start site was subcloned into the *Xho*I-*Hind*III sites of the luciferase reporter vector, pGL3-Luc (Promega), to create the HSP90alpha-Luc1430 construct. The HSP90alpha-Luc1430Mut construct was made by changing the sequence from CACGTG to CACCTG in the HSP90alpha-Luc1430 construct by using the Quick Change Site-Directed Mutagenesis Kit (Stratagene, La Jolla, CA). HepG2 cells were seeded at 1 × 10^5 ^cells/60 mm diameter plate and grown overnight. Cells were cotransfected with or without 1 μg of pcDNA3 or pcDNA3-X plasmid, 2 μg of HSP90alpha promoterluciferase reporter constructs, and 2 μg of β-galactosidase reporter plasmid (Promega) by the LipofecAMINE method (Invitrogen). Cells were cultured in 10% FBS medium for 24 h. Luciferase activity and β-galactosidase activity were assayed by using the luciferase and β-alactosidase enzyme assay system (Promega). Luciferase activity was normalized with the β-alactosidase activity in cell lysate and calculated as an average of three independent experiments.

### In vitro invasion assay

In vitro invasion assay was performed using 24-well Transwell unit with polycarbonate filters (Corning Costar, Cambridge, MA). The cells with 5 mM 10058-F4 or with RNAi treatments. were placed in the upper part of the Transwell, incubated for indicated time, fixed with methanol, and stained with hematoxylin for 10 min followed briefly by eosin. Cells in the upper chamber were removed by cotton swab and the cells that invaded through the Matrigel and were located on the underside of the filter (16 fields/filter) were counted under a microscope. The results are expressed as follows: % invasion index (the number of cells migrating through the Matrigel-coated membrane/the number of cells migrating through the uncoated control membrane) × 100. The experiment was repeated three times prepared with duplicate.

## Abbreviations

HBV: hepatitis B virus; HBx: HBV X protein; HCC: Hepatocellular carcinoma; HSP: heat shock protein; RNAi: RNA interference; siRNA: small interference RNA; GAPDH: glyceraldehyde 3-phosphate dehydrogenase; MMP: matrix metalloproteinase; RT-PCR: transcription polymerase chain reaction; EMSA: electrophoretic mobility shift assay; ELISA: enzyme-linked immunosorbent assay.

## Competing interests

The authors declare that they have no competing interests.

## Authors' contributions

WHL was responsible for most of the experimental work and drafted the manuscript. XHM and ZTQ participated in the design of this study. WTZ assisted in the statistical analysis. ZWL and ZXL participated in experiments and contributed reagents. All authors read and approved the final manuscript.
